# Draft Genome Sequence of a Chromium-Reducing Strain, *Pseudomonas fluorescens* S613, Isolated from a Chromium-Contaminated Aquifer in Los Alamos, New Mexico

**DOI:** 10.1128/genomeA.00241-17

**Published:** 2017-04-20

**Authors:** Dongping Wang, Hakim Boukhalfa, Doug S. Ware, Hajnalka E. Daligault

**Affiliations:** a Earth Systems Observations (EES-14), Earth and Environmental Sciences Division, Los Alamos National Laboratory, Los Alamos, New Mexico, USA; b Bioenergy and Biome Sciences, Biology Sciences Division, Los Alamos National Laboratory, Los Alamos, New Mexico, USA

## Abstract

In this report, a chromium-reducing bacterium, *Pseudomonas fluorescens* strain S613, was isolated from a Cr(VI)-contaminated aquifer at Los Alamos, NM, and sequenced. The size of the draft genome sequence is approximately 6.7 Mb.

## GENOME ANNOUNCEMENT

Potassium dichromate, used as an anticorrosive agent for cooling facilities at Los Alamos National Laboratory from 1956 to 1972, has resulted in significant releases of Cr(VI) to the environment. Approximately 31,000 to 72,000 kg of hexavalent chromium were discharged as effluents to the Sandia Canyon in Los Alamos, NM (1). Cr(VI) is mobile and highly toxic, therefore being of great environmental concern. Fortunately, the reduction of hexavalent chromium to the trivalent form reduces both the toxicity and mobility of chromium in the environment. The reduction of hexavalent chromium can be achieved by microbes. These bacteria reduce hexavalent chromium by certain enzymes or complexation by bacterial metabolites (2, 3). Diverse groups of bacterial species capable of reducing hexavalent chromium have been isolated and characterized. *Pseudomonas fluorescens* comprises a metabolically versatile and environmentally ubiquitous bacterial species. *P. fluorescens* strains isolated from different environments demonstrate promising chromium-resistant and metal-reducing capabilities (4–6).

In this work, we report the sequencing of a *P. fluorescens* strain which transforms chromate under aerobic conditions. The bacterium was isolated from groundwater obtained from a monitoring well located at the center of the chromium plume located in Sandia Canyon in the Los Alamos area. The strain grows on an LB plate containing 10 g/liter potassium dichromate. It also exhibits the ability to reduce Cr(VI) to Cr(III). Here, we announce the draft genome sequence of *P. fluorescens* S613. Analysis of the genome may help elucidate the molecular mechanisms that are critical for its high-level chromium tolerance and ability to reduce Cr(VI).

Genome sequencing was performed using a MiSeq Sequencer with 250-bp read chemistry (Illumina), as described previously (7–10). Briefly, total genomic DNA from *P. fluorescens* S613 was isolated from bacterial cells using the UltraClean microbial DNA isolation kit (Mo Bio, Inc., USA). The sequencing library was constructed using 12 ng of genomic DNA fragmented by the Covaris E210 instrument and prepared using NEB’s NEBNext Ultra DNA library preparation kit for Illumina. The library underwent 12 cycles of PCR and was quantified using the Qubit double-stranded DNA (dsDNA) high-sensitivity (HS) assay and quantitative PCR (qPCR). The sequencing run was set up using a version 3 MiSeq sequencing reagent kit to generate 2 × 250-bp reads.

The filtered sequences were *de novo* assembled using IDBA and Velvet and computationally shredded into 1.5-kbp overlapping shreds (11, 12). All shreds were integrated using Phrap (13). Ninety-four contigs with an average 310× genome coverage were obtained. The assembled data were annotated using an Ergatis-based (14) workflow, with minor manual curation. The genome size was found to be 6,733,904 bp, comprising 6,195 protein-coding genes. *P. fluorescens* S613 is currently explored as a hexavalent-chromium-reducing agent in the groundwater system.

### Accession number(s).

This whole-genome shotgun project of *P. fluorescens* strain S613 has been deposited at DDBJ/EMBL/GenBank under the accession no. LJXB00000000.

## References

[1] Los Alamos National Laboratory (LANL) 2007 Fate and transport modeling report for chromium contamination from Sandia Canyon. LA-UR-07-6018. Los Alamos National Laboratory, Los Alamos, New Mexico.

[2] Michel C, Brugna M, Aubert C, Bernadac A, Bruschi M 2001 Enzymatic reduction of chromate: comparative studies using sulfate-reducing bacteria. Key role of polyheme cytochromes *c* and hydrogenases. Appl Microbiol Biotechnol 55:95–100. doi:10.1007/s002530000467. 11234966

[3] Priester JH, Olson SG, Webb SM, Neu MP, Hersman LE, Holden PA 2006 Enhanced exopolymer production and chromium stabilization in *Pseudomonas putida* unsaturated biofilms. Appl Environ Microbiol 72:1988–1996. doi:10.1128/AEM.72.3.1988-1996.2006. 16517647 PMC1393198

[4] DeLeo PC, Ehrlich HL 1994 Reduction of hexavalent chromium by *Pseudomonas fluorescens* LB300 in batch and continuous cultures. Appl Microbiol Biotechnol 40:756–759. doi:10.1007/BF00173341.

[5] Bopp LH, Ehrlich HL 1988 Chromate resistance and reduction in *Pseudomonas fluorescens* strain LB300. Arch Microbiol 150:426–431. doi:10.1007/BF00422281.

[6] Chirwa EMN, Wang YT 1997 Biological reduction of hexavalent chromium by *Pseudomonas fluorescens* LB 300 in a fixed-film reactor. J Environ Eng 123:760–766. doi:10.1061/(ASCE)0733-9372(1997)123:8(760).

[7] Wang D, Han C, Dichosa A, Gleasner CD, Johnsons S, Daligault HE, Yu JM, Pierson EA, Pierson LS III 2014 Draft genome sequence of *Enterobacter cloacae* strain S611. Genome Announc 2(6):e00710-14. doi:10.1128/genomeA.00710-14. 25502660 PMC4263822

[8] Wang D, Han C, Dichosa A, Gleasner CD, Johnsons S, Daligault HE, Yu JM, Pierson EA, Pierson LS III 2013 Draft genome sequence of *Pseudomonas putida* strain S610. A seedborne bacterium of wheat. Genome Announc 1(6):e01048-13. doi:10.1128/genomeA.01048-13. 24371199 PMC3873609

[9] Wang D, Dorosky RJ, Han C, Lo CH, Dichosa A, Chain P, Yu JM, Pierson LS III, Pierson EA 2015 Adaptations genomic of a small colony variant in the biofilm of *Pseudomonas chlororaphis* 30-84. Appl Environ Microbiol 81:890–899. 25416762 10.1128/AEM.02617-14 PMC4292466

[10] Wang D, Boukhalfa H, Ware DS, Reimus PW, Daligault HE, Gleasner CD, Johnson SL, Li PE 2015 Genome sequence of a chromium-reducing strain, *Bacillus cereus* S612. Genome Announc 3(6):e01392-15. doi:10.1128/genomeA.01392-15. PMC4675937 26659672

[11] Bankevich A, Nurk S, Antipov D, Gurevich AA, Dvorkin M, Kulikov AS, Lesin VM, Nikolenko SI, Pham S, Prjibelski AD, Pyshkin AV, Sirotkin AV, Vyahhi N, Tesler G, Alekseyev MA, Pevzner PA 2012 SPAdes: a new genome assembly algorithm and its applications to single-cell sequencing. J Comput Biol 19:455–477. doi:10.1089/cmb.2012.0021. 22506599 PMC3342519

[12] Zerbino DR, Birney E 2008 Velvet: algorithms for *de novo* short read assembly using de Bruijn graphs. Genome Res 18:821–829. doi:10.1101/gr.074492.107. 18349386 PMC2336801

[13] de la Bastide M, McCombie WR 2007 Assembling genomic DNA sequences with Phrap. Curr Protoc Bioinformatics Chapter 11:Unit 11.4. doi:10.1002/0471250953.bi1104s17. 18428783

[14] Hemmerich C, Buechlein A, Podicheti R, Revanna KV, Dong Q 2010 An Ergatis-based prokaryotic genome annotation Web server. Bioinformatics 26:1122–1124. doi:10.1093/bioinformatics/btq090. 20194626

